# Dataset of mutational analysis, miRNAs targeting SARS-CoV-2 genes and host gene expression in SARS-CoV and SARS-CoV-2 infections

**DOI:** 10.1016/j.dib.2020.106207

**Published:** 2020-08-21

**Authors:** Rahila Sardar, Deepshikha Satish, Shweta Birla, Dinesh Gupta

**Affiliations:** a Translational Bioinformatics Group, International Centre for Genetic Engineering & Biotechnology (ICGEB), New Delhi, India; b Department of Biochemistry, Jamia Hamdard, New Delhi, India

**Keywords:** COVID-19, Mutation, Antiviral, miRNA, Gene expression

## Abstract

The identification of host-miRNAs targeting mutated virus genes is crucial to understand the miRNA mediated host-defense mechanism in virus infections. To understand the mechanism in COVID-19 infections, we collected genome sequences of SARS-CoV-2 with its metadata from the GISAID database (submitted till April 2020) and identified mutational changes in the sequences. The dataset consists of genes with mutation event count and entropy scores. We predicted host-miRNAs targeting the genes in the genomes and compared it with that in related viral species. We have identified 2284 miRNAs targeting MERS genomes, 2074 miRNAs targeting SARS genomes, and 1599 miRNAs targeting SARS-CoV-2 genomes, identified using the miRNA target prediction software miRanda. The host miRNAs targeting SARS-CoV-2 genes were further validated to be anti-viral miRNAs and their role in respiratory diseases through a literature survey, which helped in the identification of 42 conserved antiviral miRNAs. The data could be used to validate the anti-viral role of the predicted miRNAs and design miRNA-based therapeutics against SARS-CoV-2.

**Specifications Table**

**Table:** 

**Subject**	Bioinformatics, Genetics and Molecular Biology
**Specific subject area**	Bioinformatics
**Type of data**	Excel Files
**How data were acquired**	GISAID NCBI Genome miRBase miRanda GEO database
**Data format**	Secondary data. The secondary data Excel files have been uploaded.
**Parameters for data collection**	The Genome Detective tool was used for mutational analysis. miRanda (3.3 a version), with an energy threshold of −20 kcal/mol. Differentially expressed genes were filtered for analysis with p-value <=0.005.
**Description of data collection**	Entropy calculations were performed using metadata extracted from GISAID. miRNA targets in the virus genomes were obtained using the miRanda tool.
**Data source location**	GISAID NCBI-Genome miRBase GEO dataset ID: GSE17400 and GSE147507
**Data accessibility**	With the article
**Related research article**	Journal: R. Sardar, D. Satish, S. Birla and D. Gupta. Integrative analyses of SARS-CoV-2 genomes from different geographical locations reveal unique features potentially consequential to host-virus interaction, pathogenesis and clues for novel therapies, Heliyon. In Press.

**Value of the Data**

l The data presented here consists of mutational information such as event count and entropy score of SARS-CoV-2 genomes from different countries with the miRNA targets that could be used to understand host-virus interaction mechanisms and to design miRNA-based therapeutics.
2 The data can be useful to the virologists, biologists, bioinformaticians, pharmacologists, and biochemists interested in investigating the role of host miRNAs during SARS-CoV-2 infection.
3 The previously validated 42 antiviral miRNAs, which are also predicted to target SARS-CoV-2 genes, may be used to design experiments for miRNA-based therapeutics for COVID-19.
4 Out of these 42 miRNAs, 12 are artificial miRNAs that are experimentally validated to inhibit HIV-1 replication without any off-targets.
5 The gene expression data analysis will help to understand the host response to SARS-CoV-2 infection and may be used for comparative analysis with other viral infections.
6 Worldwide research efforts are on to control the COVID-19 pandemic. The dataset provides useful information that may be explored experimentally towards the development of alternate therapies. Hence the data could potentially make an impact on society.

## 1 Data description

The SARS-CoV-2 reference genome shares 83% aa sequence similarity with the SARS-CoV genome, the details are presented in Table S1. Table S1 also provides details of aa and nucleotide variations in the SARS-CoV-2 genome sequence with that of SARS-CoV. Mutational analysis results for the SARS-CoV-2 sequences downloaded from GISAID are stored as an excel file which consists of information of entropy scores (between 0 and 1) for the 28 SARS-CoV-2 genes, with genomic location of the mutations, in Table S2 (Sheet1 named as Entropy). The mutation event counts from different countries with corresponding SARS-CoV-2 genes are present in Table S2 (Sheet2 named as events).

The list of miRNAs targeting SARS, MERS, and SARS-CoV-2 reference genomes is available as an excel file named Table S3. The file lists the predicted targets with corresponding miRanda score, binding energy, miRNA start and end site, target start and end site, alignment length, similarity, and identity percentage. These miRNAs were compared with a list of experimentally proven anti-viral miRNAs (against any known virus) from the literature. We found 42 such miRNAs, the details of these miRNAs with miRBase accession ID, miRNA sequence, viral target with their UniProt ID and PubMed accession number (PMID) is available in the Table S3, sheet3 (named as 42_miRNA).

The gene expression datasets representing virus-infected human cell lines were retrieved from the GEO database. We downloaded the NCBI GEO SARS-CoV microarray dataset (GEO ID GSE17400) and SARS-CoV-2 RNA sequencing dataset (GEO ID GSE147507) (Table S4), analysis sample collected at 24 hr in both the datasets. The SARS-COV-2 data, generated from A459 and NHBE cell lines, showed 131 and 606 differentially expressed genes respectively. During SARS-CoV infection, 2796 genes were differentially expressed (Table S4).

## 2 Experimental design, materials and methods

High coverage and complete SARS-CoV-2 genome sequences and its corresponding metadata, submitted till 15th April 2020, were retrieved from the GISAID database [1]. The SARS-CoV (NC_004718.3) and MERS (KC164505.2) genomes were downloaded from the NCBI genome database and compared with SARS-CoV-2 reference genome sequence (NC_045512.2; sequence from Wuhan, China). Complete, high coverage SARS-CoV-2 genomes from the GISAID database were subjected to mutational analysis using Genome Detective Coronavirus subtyping Tool (version 1.1.3) [2]. To remove redundancy in the sequences, the genome nucleotide sequences that share 99.99% similarity with the reference genome (NC_004718.3) were excluded from further analysis. We downloaded the sequences of all the available 2654 mature human miRNAs from the miRBase (Release 22.1) [3]. Additionally, we also surveyed the literature to identify experimentally validated antiviral miRNAs, including artificial miRNAs. Comparing our human miRNA target predictions for the three viruses and the list of antiviral miRNAs (against any known virus) from the literature survey, we found 42 miRNAs to be common. Twelve out of these are artificial miRNAs, however these also target the three viral genomes studied by us. These miRNAs were used to identify potential miRNA target sites in the virus genome sequences, using miRanda (3.3 a version) [4], with an energy threshold of −20 kcal/mol, a threshold used in other studies too [5]. SARS-CoV (NC_004718.3), MERS (NC_019843.3), and SARS-CoV-2 isolate from Wuhan (NC_045512.2) were used as target viral genomes. Differentially expressed genes with the p-value <=0.005 for SARS-CoV and SARS-CoV-2 expression data were used for the analysis. The details of the analysis of these datasets are available from Sardar et al. [6].

## Ethics statement

The dataset is based on bioinformatic analysis; therefore, no animal has been used and/or harmed in the present investigation.

## Declaration of Competing Interest

The authors declare that they have no known competing financial interests or personal relationships which have, or could be perceived to have, influenced the work reported in this article.

## Appendix Supplementary materials

Image, application 1
